# Evolutionary adaptation of CCD4 enzymes in *Buddleja alternifolia* for crocetin biosynthesis

**DOI:** 10.3389/fpls.2026.1916011

**Published:** 2026-08-20

**Authors:** Eduardo Parreño, Lucía Morote, Alberto J. López Jiménez, Elena Moreno-Giménez, Ángela Rubio-Moraga, Oussama Ahrazem, Lourdes Gómez-Gómez

**Affiliations:** 1 Instituto Universitario de Biotecnología, Agronomía y Sostenibilidad de la Universidad de Castilla-La Mancha (IBAS), Albacete, Spain; 2 Université Jean Monnet Saint-Etienne, Centre National de la Recherche Scientifique, Laboratoire de Biotechnologies Végétales Appliquées aux Plantes Aromatiques et Médicinales, Unité Mixte de Recherche, Saint-Etienne, France

**Keywords:** apocarotenoids, carotenoid cleavage dioxygenase, CCD4 evolution, crocetin, gene duplication, specialized metabolism

## Abstract

**Introduction:**

Carotenoid cleavage dioxygenase 4 (*CCD4*) enzymes play central roles in carotenoid turnover and apocarotenoid biosynthesis in plants. Despite their importance, the evolutionary mechanisms underlying diversification of *CCD4* catalytic functions remain poorly understood. This study investigated the CCD gene family in *Buddleja alternifolia*, with particular emphasis on the expansion and functional evolution of the *CCD4* subfamily.

**Methods:**

A genome-wide identification and comparative analysis of CCD genes were performed in *B. alternifolia*. Genomic organization, phylogenetic relationships, and syntenic patterns were analyzed to investigate gene family expansion. Functional characterization of 11 Ba*CCD4* paralogs was conducted through biochemical assays, while structural analyses were used to identify sequence features associated with differences in substrate cleavage specificity. Gene expression profiling was performed to assess patterns of tissue-specific regulation.

**Results:**

Twenty-three CCD genes were identified, including 12 *CCD4* paralogs, representing one of the largest *CCD4* expansions reported within Lamiales. Syntenic and genomic analyses revealed that recent tandem duplication events, particularly within a *CCD4*-rich region on chromosome 10, were the primary drivers of this expansion. The presence of pseudogenes in the same region supported an ongoing birth-and-death evolutionary process. Functional analyses demonstrated extensive biochemical diversification among Ba*CCD4* enzymes despite their high sequence similarity. Several paralogs catalyzed asymmetric carotenoid cleavage leading to citraurin production, whereas two paralogs, KAG8367281 and KAG8375220, exhibited symmetric zeaxanthin cleavage activity, producing crocetin dialdehyde, the direct precursor of crocins. Notably, these crocetin-producing enzymes belonged to closely related paralogous pairs whose counterparts displayed distinct cleavage specificities, indicating rapid neofunctionalization after duplication. Structural analyses suggested that subtle sequence variations, including indels affecting loop regions adjacent to the substrate access channel, may underlie changes in regioselectivity. Expression profiling further revealed tissue-specific expression patterns consistent with functional divergence among paralogs.

**Discussion:**

These findings indicate that crocetin-forming activity in *B. alternifolia* likely evolved through progressive modifications of ancestral *CCD4* functions rather than through a single evolutionary event. The remarkable expansion and diversification of the *CCD4* subfamily provide evidence for the role of gene duplication and neofunctionalization in shaping carotenoid cleavage specificity. Collectively, this work establishes *B. alternifolia* as a valuable model for investigating the molecular evolution of *CCD4* enzymes and the emergence of specialized apocarotenoid metabolism in plants.

## 1 Introduction

Plant specialized metabolism emerged from the land colonization by ancient plants, becoming diversified along with plant evolution. A variety of secondary metabolites, from pigments and flavors to volatiles, mediate a broad network of interspecies interactions that allow plants to adapt to the array of natural ecosystems (Weng et al., 2012). To date, over one thousand naturally occurring carotenoids have been identified, and significant progress has been made in elucidating their biosynthetic pathways at the molecular level (Yabuzaki, 2017; Jacob-Lopes et al., 2018). This remarkable chemical diversity reflects the strong and often fluctuating selective pressures imposed by ecological interactions, which continuously shape secondary metabolic networks. Several studies have shown that rates of evolution are greater for genes involved in plant secondary metabolism than in primary metabolism (Scossa and Fernie, 2020). This observation raises important questions regarding the molecular evolution and adaptive diversification of plant specialized metabolic processes (Carretero-Paulet and Fares, 2012). One notable example is the carotenoid cleavage dioxygenase (CCD) family, which catalyzes the formation of apocarotenoids and has undergone lineage-specific expansion of several subfamilies in some plant species. CCDs participate in the biosynthesis of diverse apocarotenoids, including the hormone ABA, volatile compounds such as β-ionone, pigments such as crocins and bixin, and signaling molecules such as strigolactones (Moreno et al., 2021). This evolutionary diversification has been accompanied by substantial functional specialization, resulting in CCD subfamilies with distinct biochemical activities and biological roles across the plant lineage. The CCD family in plants is classified into six groups: CCD1, CCD2, CCD4, CCD7, CCD8, and CCD10. The CCD1 enzymes, all cytosolic and soluble enzymes, cleave a variety of both linear and cyclic carotenoids at different positions to produce apocarotenoids, but are also involved in the cleavage of apocarotenoids. Therefore, CCD1 enzymes are most probably involved in the degradation of apocarotenoid compounds (Auldridge et al., 2006; Ilg et al., 2010). CCD2 enzymes are found only in certain monocotyledonous species and recognize and cleave specifically zeaxanthin at 7,8:7′,8′ positions to form crocetin dialdehyde and 3-hydroxy-β-cyclocitral (Frusciante et al., 2014; Ahrazem et al., 2016b; Fang et al., 2020; Morote et al., 2025a). Like CCD2, CCD7 and CCD8 catalyze highly specific carotenoid cleavage reactions and are involved in the biosynthesis of carlactone, the universal precursor of strigolactones, from 9-cis-β-carotene (Alder et al., 2012). Related to the strigolactone pathway are as well the CCD10 enzymes, whose first member was identified and characterized in rice (Wang et al., 2019). By contrast to these enzymes, the CCD4 enzymes, localized in plastids, cleave carotenoids asymmetrically or symmetrically at 7,8:7′,8′ contributing to coloration in plant tissues such as *Citrus* peels (Ma et al., 2013; Rodrigo et al., 2013), *Buddleja davidii* flowers (Ahrazem et al., 2017) or gardenia fruits (Xu et al., 2020), or can cleave carotenoids asymmetrically or symmetrically at 9,10:9′,10′ positions, resulting in volatile production in flowers and fruits (Rubio et al., 2008; Huang et al., 2009; Morote et al., 2023), and contributing to carotenoids degradation (Ohmiya et al., 2009; Soares et al., 2011; Zhang et al., 2015; Wang et al., 2018).

The broad substrate specificity and cleavage promiscuity observed in these enzymes may help explain the evolutionary processes underlying the extensive structural diversity of plant apocarotenoids. New CCDs activities may have emerged through early gene duplication, followed by mutations that broadened substrate selection and flattened activation barriers of their catalyzed reactions. A special case among the CCDs is the CCD4 family which, as discussed above, compiles the highest number of members with different specificities at the level of substrate recognition and double bond cleavage. Among the apocarotenoids produced through the enzymatic activity of CCD4, crocetin stands out as a particularly significant compound due to its well-documented biological activities and growing body of evidence in the biomedical literature (Guo et al., 2022), making it a molecule of considerable interest. CCD4 enzymes involved in crocetin biosynthesis have been identified in *B. davidii* (Ahrazem et al., 2017), gardenia (Xu et al., 2020), *Verbascum sinuatum* and *V. giganteum* (Morote et al., 2024) and in *Nyctanthes arbor-tristis* (Morote et al., 2025b). Despite lineage-specific expansion of the CCD4 gene family in crocetin-producing species, only a subset of paralogs has evolved crocetin-forming activity. In *B. davidii* and *Verbascum* spp., two of the three characterized CCD4 enzymes catalyze the 7,8:7′,8′ cleavage of carotenoids, whereas this activity has been demonstrated for only one of the five CCD4 paralogs in *G. jasminoides* and one of the eight in *N. arbor-tristis*.

To clarify the process of emergence of crocetin biosynthesis in plants, this study focused on the *CCD* genes present in the genome of *Buddleja*. Recently, the genome of *Buddleja alternifolia* has been revealed (Ma et al., 2021) allowing the identification of genes encoding for CCD enzymes in general and CCD4 in particular. The phylogenetic relationship of these CCDs to previously reported CCDs from *B. davidii* was also analyzed, and the enzymatic activity of 11 of them assayed using different carotenoid substrates, to determine their cleavage activities and substrate preferences. In conclusion, our study showed that the combined identification, phylogenetic analysis, sequence comparison, structural characterization, and enzymatic evaluation of the CCD4 gene family in *B. alternifolia* provide a comprehensive framework for understanding the evolutionary origin of crocetin biosynthesis in dicotyledonous plants. The results indicate that diversification within the CCD4 subfamily, driven by sequence variation and associated structural changes, gave rise to enzymes with distinct catalytic properties and substrate specificities. These findings support the hypothesis that the emergence of crocetin biosynthesis resulted from the functional specialization of members of the CCD gene family during plant evolution.

## 2 Materials and methods

### 2.1 Databases for genomic, cDNA, and protein sequences

In this study, the genomic data from *B. alternifolia* was used for genome-wide identification of *CCDs*. The genome from *B. alternifolia* has been completely sequenced and annotated. The sequence data was downloaded from the NCBI genome database (https://www.ncbi.nlm.nih.gov/genome), BioProject accession PRJNA577174.

### 2.2 Identification of CCDs by BLASTP searches

Candidate carotenoid cleavage dioxygenase (CCD) proteins were identified using a combined conserved-domain and sequence similarity approach. First, all predicted protein sequences from the genome were screened for the presence of the conserved carotenoid oxygenase domain, including Pfam accession PF03936 and the PLN02258 superfamily annotation. To avoid missing divergent CCD family members, standalone BLASTP searches were performed against the predicted protein database using representative Arabidopsis CCD protein sequences as queries, including CCD1 (NP191911), CCD4 (NP193652), CCD7 (NP001318427), CCD8 (NP001329787), and NCED (QAP15631), together with the CCD10 protein from *Solanum lycopersicum* (XP025883852). BLASTP searches were conducted using an E-value cutoff of 1 × 10^-^^5^, with a minimum query coverage of 50% and a minimum amino acid identity of 30%. Redundant hits were removed, and all candidate proteins were further verified for the presence of the conserved carotenoid oxygenase domain using the Pfam database and the NCBI Conserved Domain Database (CDD). Only sequences containing a complete conserved carotenoid oxygenase domain were retained as bona fide CCD candidates. The final classification of CCD family members into CCD1, CCD4, CCD7, CCD8, NCED, and CCD10 subfamilies was based on sequence similarity to experimentally characterized CCD proteins, conserved domain architecture, and their phylogenetic relationships with known CCD proteins from Arabidopsis and tomato as representative plant species.

### 2.3 Phylogenetic tree construction and CCDs classification

Amino acid sequences were aligned using the MAFFT online server (version 7; https://mafft.cbrc.jp/alignment/server/) (Katoh et al., 2019) with the Auto strategy and default parameters. The resulting multiple sequence alignment was used to infer phylogenetic relationships by the maximum-likelihood (ML) method implemented in MEGA11 (Tamura et al., 2021). The Jones-Taylor-Thornton (JTT) substitution model was used for amino acid sequence evolution. Initial trees for the heuristic search were generated automatically using the Neighbor-Joining and BioNJ algorithms based on pairwise distances estimated under the JTT model, and the topology with the highest log-likelihood value was selected. The robustness of the inferred phylogeny was evaluated by bootstrap analysis with 1,000 replicates. Sites containing gaps or missing data were excluded using the complete deletion option, and uniform substitution rates among sites were assumed. The family classification was carried out according to the Arabidopsis CCD sequences and the tomato CCD10.

### 2.4 Detection of duplicated and tandem arrangement sequences

Tandemly duplicated genes were identified by their sequence similarity and chromosomal localization according to (Jiang et al., 2009). In brief, a tandemly duplicated gene was identified through comparison with its putative parental gene. To satisfy the identification criteria, the gene must meet the following conditions: (1) a minimum of 30% of its sequence must align with the parental gene sequence, as determined by BLASTP searches using an E-value threshold of 0.01; (2) the amino acid sequences of the two genes must share at least 70% identity; (3) the genes must be located within 10 genes of each other and positioned within 100 kb for genomes smaller than 200 Mb, or within 350 kb for genomes larger than 200 Mb.

### 2.5 Pseudogenes detection

Putative pseudogenes were identified through manual inspection of the predicted gene models and their encoded protein sequences. Candidate pseudogenes were defined based on the presence of one or more of the following characteristics: (i) premature stop codons resulting in truncated open reading frames, (ii) frameshift mutations caused by insertions or deletions that disrupted the coding sequence, and/or (iii) the absence or severe truncation of the conserved carotenoid cleavage dioxygenase (CCD) domain compared with functional CCD4 homologs. Gene structures and predicted protein sequences were compared with intact CCD4 genes from the same genome and from closely related species to distinguish functional genes from putative pseudogenes.

### 2.6 Analysis of *Buddleja alternifolia CCD4* expression across tissues

To analyze the expression of *CCD4* genes in *B. alternifolia*, annotated transcript sequences from the reference genome BAv1.1 (GenBank accession GCA_019426215.1) were used as reference transcriptome. Publicly available RNA-seq datasets from *Buddleja alternifolia* BioProject PRJNA577174 were retrieved from the NCBI Sequence Read Archive. The datasets included fruit (SRR10305034; BioSample SAMN13022357), leaf (SRR10305045; SAMN13022356), stem (SRR10305056; SAMN13022355), and flower (SRR10305067; SAMN13022354) samples (**Supplementary Table 1**). RNA-seq reads were mapped against the reference transcriptome using Bowtie2 (Langmead and Salzberg, 2012). Transcript abundance was subsequently estimated with Salmon using the Bowtie2 alignments as input. Expression values were normalized as transcripts per million (TPM). The TPM values corresponding to annotated *CCD4* transcripts were extracted and used to compare tissue-specific expression patterns. Expression data were represented as TPM values across the analyzed tissues.

### 2.7 Microsynteny analysis of CCD4 genomic regions

Local microsynteny around *CCD4* loci was examined in *B. davidii*, *B. alternifolia*, *Vitis vinifera*, and *Amborella trichopoda*. The genome assemblies used were *A. trichopoda* AMTR1.0 (RefSeq GCF_000471905.2), *V. vinifera* ASM3070453v1 (RefSeq GCF_030704535.1), *B. alternifolia* BAv1.1 (GenBank accession GCA_019426215.1), and *B. davidii* daBudDavi1.hap1.2 (GenBank accession GCA_965641825.2). Genomic regions containing *CCD4* homologues were extracted from *A. trichopoda* unplaced scaffold NW_006494910.1; *B. alternifolia* chromosomes 14 (CM033403), 16 (CM033405), 10 (CM033399), and 5 (CM033394); *V. vinifera* chromosome NC_081806.1; and *B. davidii* chromosomes 16 (OZ282516) and 12 (OZ282512). Gene models for the analyzed *B. davidii* regions were predicted using AUGUSTUS (Stanke et al., 2006). Microsynteny was analyzed using Clinker through the CEGECAT server (Van Den Belt et al., 2023). Homologous relationships between genes across the compared genomic regions were inferred using a minimum amino acid sequence identity threshold of 40%.

### 2.8 Biochemical characterization

For each *CCD* gene selected for biochemical characterization, the encoded protein was first analyzed using TargetP (https://services.healthtech.dtu.dk/service.php?TargetP-2.0) to predict the putative transit peptide. Then, with the sequence for putative transit peptide removed and a codon for Met added to the beginning of the coding sequence, a complementary DNA for each *CCD* was synthesized and cloned into pThio-Dan1 vector by infusion (Takara). *Escherichia coli* strain BL21(DE3) was used as the heterologous host for carotenoid cleavage activity assays. Competent cells were carrying the plasmids Pac-LYC, Pac-ZEAX, and Pac-BETA (Addgene) to enable the biosynthesis of lycopene, zeaxanthin, and β-carotene, respectively. These bacterial cells were subsequently transformed with either the expression plasmid containing the different CCD4 enzymes or the empty vector pThio-Dan1, which served as a negative control. Transformed cells were cultured overnight at 37 °C in 3 mL of Luria-Bertani (LB) medium supplemented with ampicillin (100 µg mL^-^¹) and chloramphenicol (60 µg mL^-^¹) with shaking at 200 rpm. For activity assays, overnight cultures were used to inoculate Terrific Broth (TB; Invitrogen) supplemented with ampicillin (50 µg mL^-^¹) and chloramphenicol (30 µg mL^-^¹), and cultures were grown at 30 °C with shaking at 200 rpm. Recombinant protein expression was induced by the addition of L-arabinose to a final concentration of 0.8% (w/v). Immediately after induction, n-dodecane was added to a final concentration of 10% (v/v), following the previously described protocol (Morote et al., 2025b). The products were identified using HPLC analyses as previously described (Moreno-Giménez et al., 2026). Briefly, following overnight cultivation the n-dodecane layer containing the carotenoid cleavage products was carefully recovered and centrifuged at 10,000 × g for 10 min to eliminate residual cell debris. The clarified organic phase was subsequently used directly for apocarotenoid analysis by high-performance liquid chromatography coupled to a diode-array detector (HPLC-DAD; Agilent 1100 Series, Agilent Technologies). Separation was performed on a YMC C30 reverse-phase column (250 × 4.6 mm, 5 μm; Waters, Milford, MA, USA), and chromatograms were monitored at 440 and 300 nm. The chromatographic mobile phase consisted of solvent A (methanol, 98:2, v/v), solvent B (methanol, 95:5, v/v), and solvent C (100% methyl tert-butyl ether). Elution was carried out at a flow rate of 1.0 mL min^-^¹ and a column temperature of 40 °C using the following gradient program: 80% A and 20% C at the start of the run; a linear gradient to 60% A and 40% C during the first 3 min; at 4 min the mobile phase was adjusted to 60% B and 40% C; this was followed by a linear gradient to 100% C at 12 min. Initial chromatographic conditions were restored at 13 min, and the column was re-equilibrated for 10 min before the next injection. Apocarotenoid products were identified based on their retention times and UV-visible absorption spectra and by comparison with authentic or reference standards. An authentic crocetin dialdehyde standard (sigma-Aldrich) was used to confirm the identity of crocetin dialdehyde. Because a commercial β-citraurin standard was not available, β-citraurin was identified by comparison with the reaction product generated by the Citrus CitCCD4b enzyme, which specifically cleaves zeaxanthin to produce β-citraurin under the same chromatographic conditions. Product identities were assigned by matching retention times and absorption maxima (λmax) with those of the corresponding standards or reference reaction products. All experiments were performed using three independent biological replicates.

### 2.9 Bioinformatic analysis of protein sequences

The prediction of the location of all CCD4 proteins was done with DeepLoc-1.0 (https://services.healthtech.dtu.dk/services/DeepLoc-1.0/). The proteins were modelled using Phyre2 (Powell et al., 2025) (https://www.sbg.bio.ic.ac.uk) the normal modelling mode. Tunnels leading to the catalytic iron atom were identified and visualized using the MOLEonline server (https://mole.upol.cz) (Raček et al., 2025) using default parameters. Structural visualization, hydrophobicity surface analysis, and cross-sectional views of the substrate-entry tunnel were performed using UCSF Chimera X program (https://www.cgl.ucsf.edu/chimera/) (Meng et al., 2023).

## 3 Results

### 3.1 *CCD* genes in the genome of *B. alternifolia*

**Figure 1:**
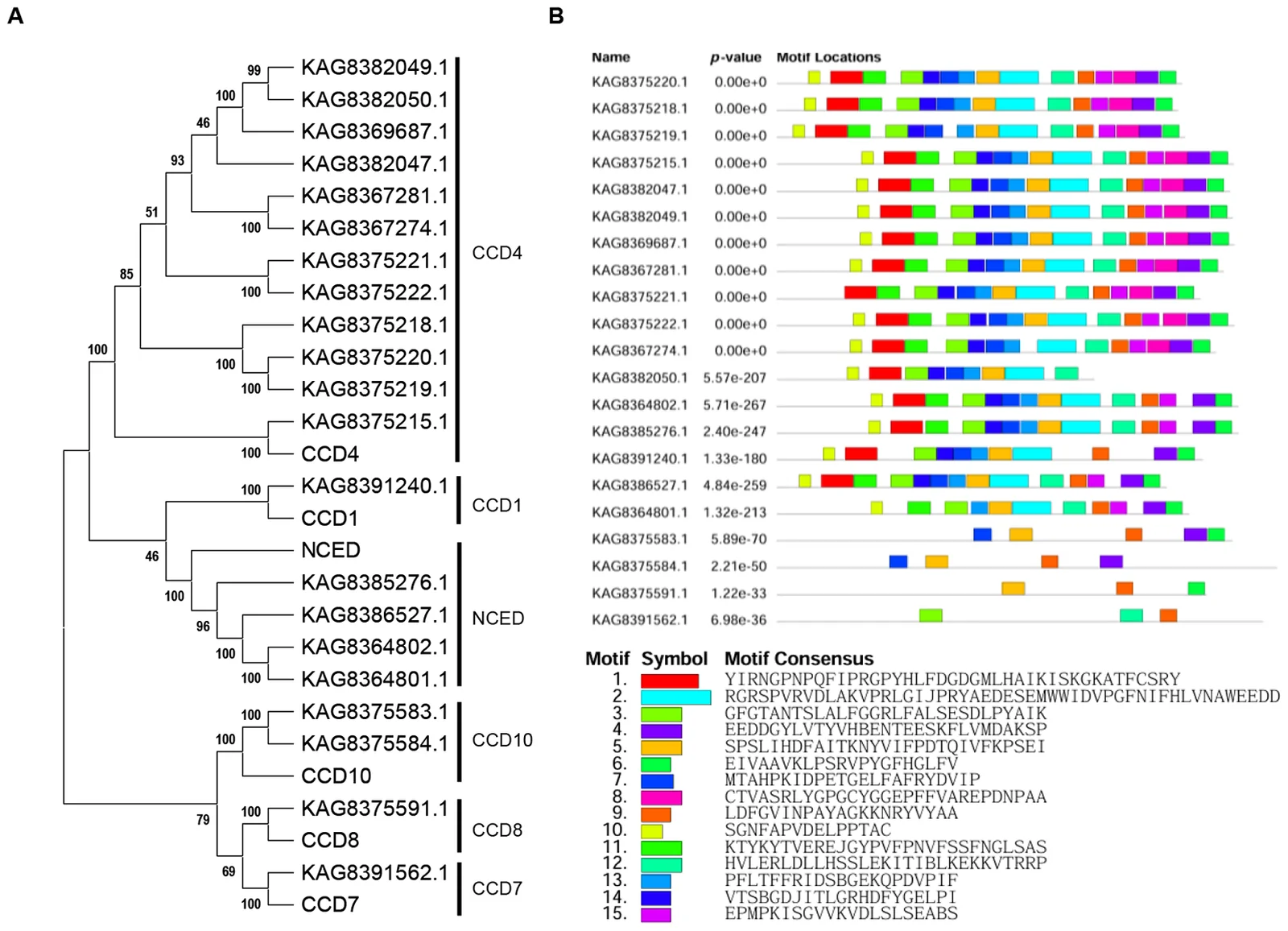
Phylogenetic analysis of carotenoid cleavage dioxygenase (CCD) proteins from *Buddleja alternifolia* and representative plant species, and conserved protein motifs of *B. alternifolia* CCD proteins. **(A)** Unrooted phylogenetic tree inferred using the Neighbor-Joining method implemented in MEGA11 based on the amino acid sequences of 27 CCD proteins. Evolutionary distances were calculated using the Jones–Taylor–Thornton (JTT) substitution model. Numbers at the nodes indicate bootstrap support values based on 1,000 replicates. **(B)** Distribution of conserved protein motifs identified in *B. alternifolia* CCD proteins using the MEME Suite. Different colors represent the 15 conserved motifs (shown below the panel), and the corresponding amino acid sequences of each motif are provided.

Following a previously developed strategy for the identification of the *CCD* members in *Paulownia tomentosa* (Morote et al., 2023), we found a total of 23 genes in the genome of *B. alternifolia* (GenBank GCA_019426215.1), two of them exhibited characteristics of pseudogenes (*Bualt10G0077300* and *Bualt0077400*), including multiple repetitive regions and incomplete coding sequences that prevented the prediction of full-length proteins. Although BLASTP analysis of the translated partial sequences indicated that both *loci* shared the highest sequence similarity with Arabidopsis CCD4, their incomplete protein sequences precluded reliable phylogenetic analysis. Therefore, a phylogenetic tree was constructed using the amino acid sequences of the identified full-length CCD proteins together with the Arabidopsis CCD1, CCD4, CCD7, CCD8, and NCED proteins, and the tomato CCD10 protein as references. Homologs for all the subfamilies were identified (**Figure 1**). Unique representatives were found for CCD1, CCD7 and CCD8. Two representatives were found for CCD10, and 4 for NCED. The biggest subfamily was the CCD4, with a total of 12 complete CCD4 members (**Figure 1A** and two truncated coding sequences (**Table 1**). MEME (Motif-based sequence analysis tools) (Bailey et al., 2015) analysis of CCD proteins identified 15 conserved motifs, and their distributions are shown in **Figure 1B**. Conservative motif analysis showed that most motifs clustered in the same phylogenetic taxon share a common motif composition.

**Table 1:** Characteristics of CCD4 genes and proteins of *B. alternifolia*.

Protein ID	Gene length	CDS length	Protein length	Intron number	Chr n°	% identity BdCCD4.1	% identity BdCCD4.2	% identity BdCCD4.3
KAG8382047.1	4880	1764	587	1	5	58.72	–	56.69
KAG8382049.1	3948	1776	591	1	5	61.04	–	–
KAG8382050.1	1371	1236	411	1	5	–	–	–
KAG8375215.1	3538	1779	592	1	10	53.9	98.14	57.68
KAG8375216.1	14950	975	324	3	10	–	–	–
KAG8375217.1	3706	654	214	3	10	–	–	–
KAG8375218.1	3867	1563	520	3	10	61.99	–	–
KAG8375219.1	7459	1590	529	5	10	57.98	–	83.21
KAG8375220.1	9881	1578	525	3	10	59.28	–	88.42
KAG8375221.1	8279	1650	549	2	10	58.97	–	–
KAG8375222.1	5489	1781	593	1	10	54.08	–	56.02
KAG8369687.1	2358	1782	593	1	14	60.00	–	–
KAG8367281.1	3086	1740	579	2	16	94.31	–	–
KAG8367274.1	3085	1710	569	2	16	89.31	–	–

### 3.2 The CCD4 sub-family in *B. alternifolia*

**Figure 2:**
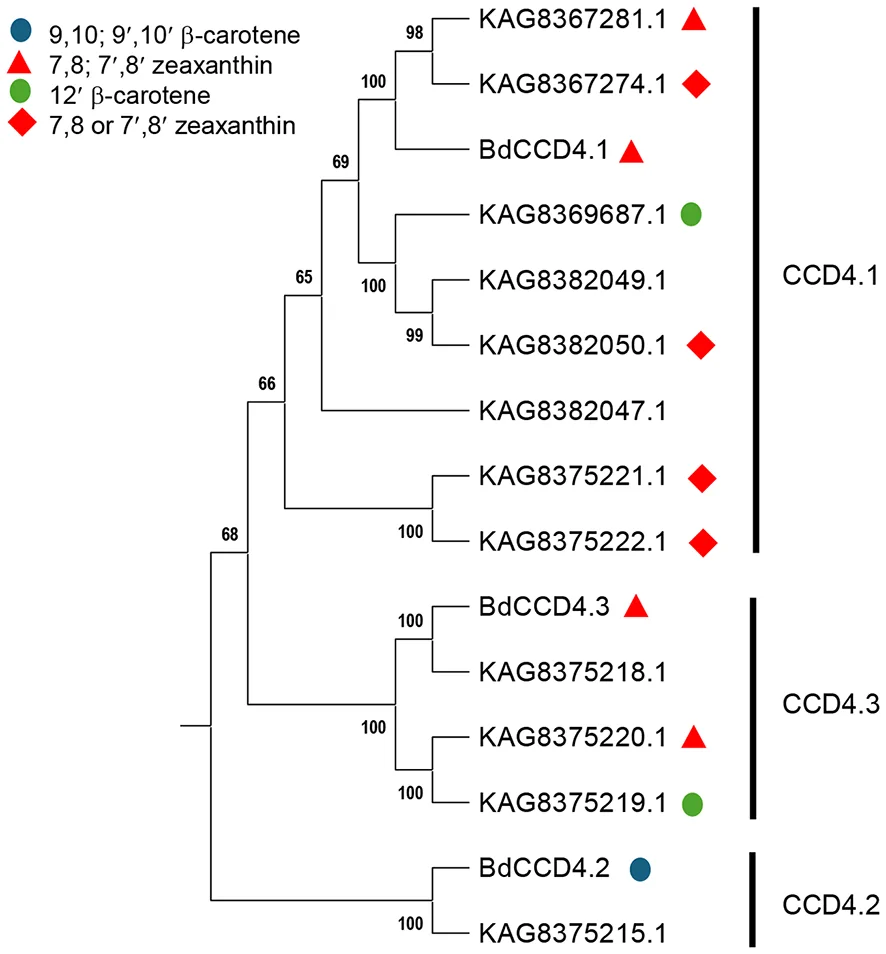
| Phylogenetic analysis of the CCD4 subfamily from *Buddleja alternifolia* and its relationship to functionally characterized CCD4 enzymes from *Buddleja davidii*. The phylogenetic tree was constructed using the Neighbor-Joining method based on the Jones–Taylor–Thornton (JTT) substitution model. Numbers at the internal nodes indicate bootstrap support values (%). The three major CCD4 clades are indicated on the left side of the tree. Members of the CCD4.1 and CCD4.3 clades were selected for functional characterization of carotenoid cleavage activity. Colored symbols adjacent to each sequence name denote the experimentally determined substrate specificity of the corresponding CCD4 enzyme toward lycopene, β-carotene, or zeaxanthin. The absence of a symbol indicates that no cleavage activity was detected with any of the tested substrates. The inset in the upper left illustrates the colored symbols used to represent each carotenoid substrate together with the corresponding cleavage position catalyzed by the enzyme.

Previously, three CCD4 enzymes from *B. davidii* were identified and their activities characterized, with BdCCD4.1 and BdCCD4.3 involved in crocetin biosynthesis with a 7,8:7′,8′ cleavage activity, while BdCCD4.2 showed a 9,10:9′,10′ activity (Ahrazem et al., 2017). Using these three enzymes from *B. davidii* as baits, BdCCD4.1, BdCCD4.2 and BdCCD4.3, we classified the 12 genes from *B. alternifolia* encoding for CCD4 enzymes with identities ranging from 94% to 60%. The CCD4 enzymes were distributed in three major clusters (**Figure 2**). 8 enzymes were in the CCD4.1 cluster, 3 in the CCD4.3 cluster and 1 in the CCD4.2 cluster (**Figure 2**).

**Figure 3:**
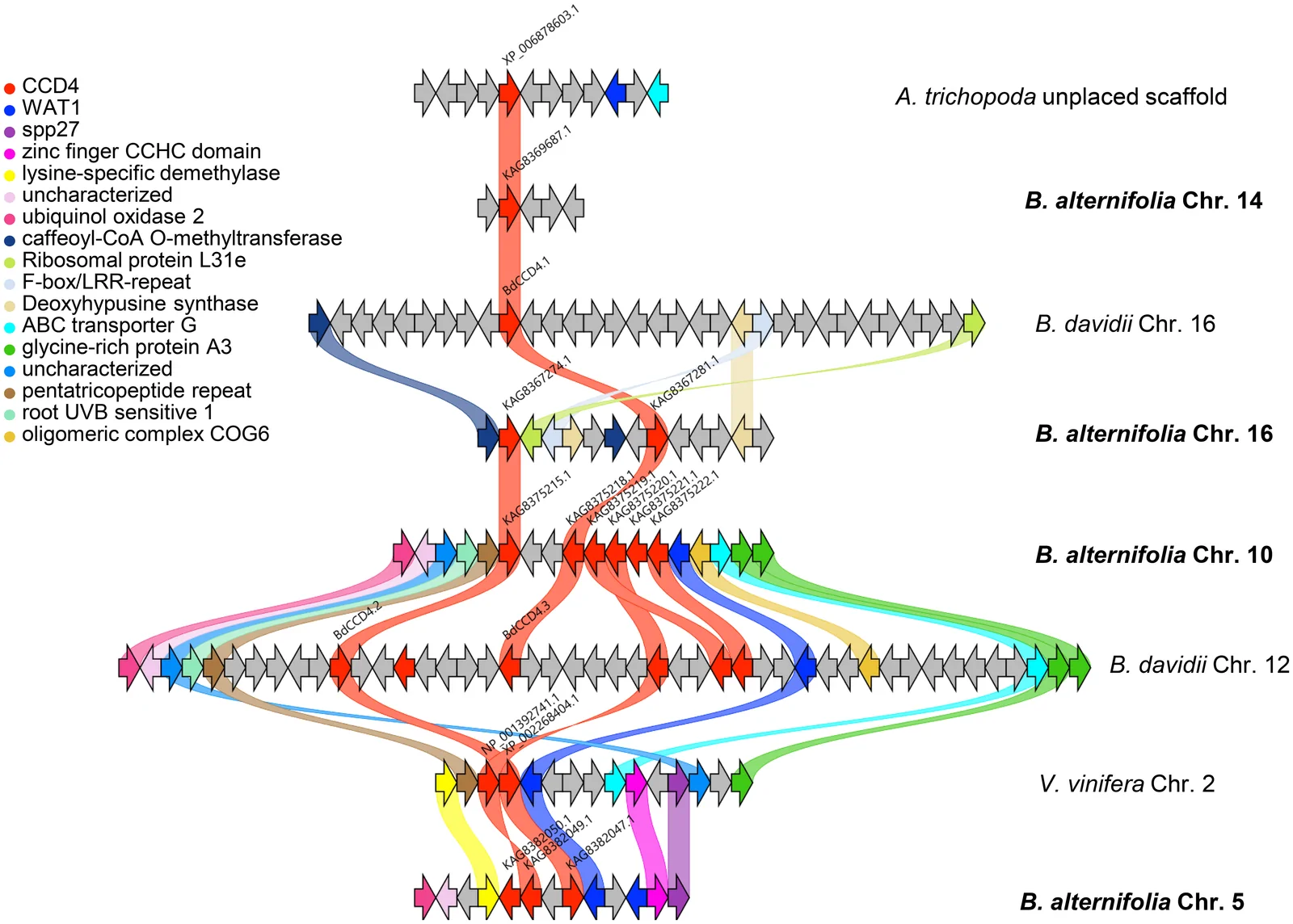
Local microsynteny comparison of genomic regions containing *CCD4* genes in *B. davidii*, *B. alternifolia*, *V. vinifera*, and *A. trichopoda*. The analysis was performed using Clinker through the CEGECAT server. Arrows indicate predicted genes and their transcriptional orientation, while colored links represent homologous relationships between genes across the compared regions. To simplify the visualization, only best-hit relationships are colored. Only genes corresponding to *CCD4* homologues are labelled. The genomic regions shown correspond to *A. trichopoda* unplaced scaffold NW_006494910.1; *B. alternifolia* chromosomes 14 (CM033403), 16 (CM033405), 10 (CM033399), and 5 (CM033394); *V. vinifera* chromosome NC_081806.1; and *B. davidii* chromosomes 16 (OZ282516) and 12 (OZ282512). The *B. davidii* gene annotation was generated using AUGUSTUS.

The genes encoding for these 12 enzymes were distributed in four out of the 19 chromosomes of *B. alternifolia*, in chromosomes 5, 10, 14 and 16 (**Table 1**; **Figure 3**). A total of 6 genes were located on Chr10, excluding the two pseudogenes (**Table 1**; **Figure 3**). Interestingly, in Chr10 the genes encoding for proteins KAG8375221 and KAG8375222 are separated by less than 10Kb, and showed 98.56% identity, and similar situation is found for proteins KAG8375219 and KAG8375220, which showed 93.12% identity, suggesting recent duplication events. The same situation was observed for KAG8367274 and KAG8367281 encoded for the only two genes present on Chr16, with a 93.26% identity. Finally, on Chr5 three genes were located, KAG8382049 and KAG8382050, more closely related, and KAG8382047 (**Table 1**; **Figure 3**). All the genes contained introns in a variable number, ranging from 1 to 5 (**Table 1**). The syntenic relationships of *BaCCD4* genes were performed with homologs in three plant species, including *B. davidii*, *V. vinifera* and *A. trichopoda*. In *A. trichopoda*, only one homolog gene was present, while two were present in chromosome 2 of *V. vinifera* (**Figure 3**). A total of 7 homologs genes, including 1 in chromosome 16 and 6 in chromosome 12, were identified *B. davidii* (**Figure 3**). This analysis of gene content and gene density among the four different species revealed that *B. alternifolia* showed the highest number of genes, with a total of 12 sequences (**Figure 3**). Collectively, these genomic features, including the presence of closely linked duplicated genes, pseudogenes, and the observed syntenic relationships, are consistent with tandem duplication events and suggest that the BaCCD4 gene family has evolved through a birth-and-death evolutionary process.

All the amino acid sequences from *B. alternifolia* and the three CCD4 proteins from *B. davidii*, were used to construct a phylogenetic tree (**Figure 2**). The amino acid sequences distributed in three well supported clusters, corresponding to the ones where the three *BdCCD4* genes were distributed (**Figure 2**). The CCD4.1 cluster was constituted by 8 members, whose genes are distributed in three chromosomes (5, 14 and 16). The CCD4.2 cluster contained only one representative, located on chromosome 10, separated from the CCD4.1 member by 122,408 bp, and from the CCD4.3 members by 74,571bp. The two identified pseudogenes were in this region (**Supplementary Figure S1**), the 324 amino acid residues of sequence KAG8375216 encoded by Bualt10G0077300, showed the highest identity (86.79%) to KAG8375218, while the 220 amino acid residues of sequence KAG8375217 encoded by Bualt0077400, showed the highest identity (73.79%) to KAG8375220, both belong to the CCD4.3 cluster. The CCD4.3 cluster contained 3 proteins, all located on chromosome 10, besides the truncated ones (**Figure 2**). However, this distribution changed when the sequences of the recently characterized *Verbascum* species were added to the analyses (**Figure 3**). A total of 6 proteins, KAG8375220, KAG8375219, KAG8375218, KAG8382047, KAG8375221, and KAG8375222, are enclosed in cluster CCD4.3, and sequences KAG8382049, KAG8382050, KAG8369687, KAG8367281, and KAG8367274 were inside the CCD4.1 cluster (**Figure 3**).

### 3.3 The expression patterns of the *BaCCD4* sub-family

Transcriptome reads from four *B. alternifolia* organs, fruit (SRR10305034), leaf (SRR10305045), stem (SRR10305056), and flower (SRR10305067), obtained from BioProject PRJNA577174, were mapped to the gene loci to estimate gene expression as transcripts per million (TPM).

**Figure 4:**
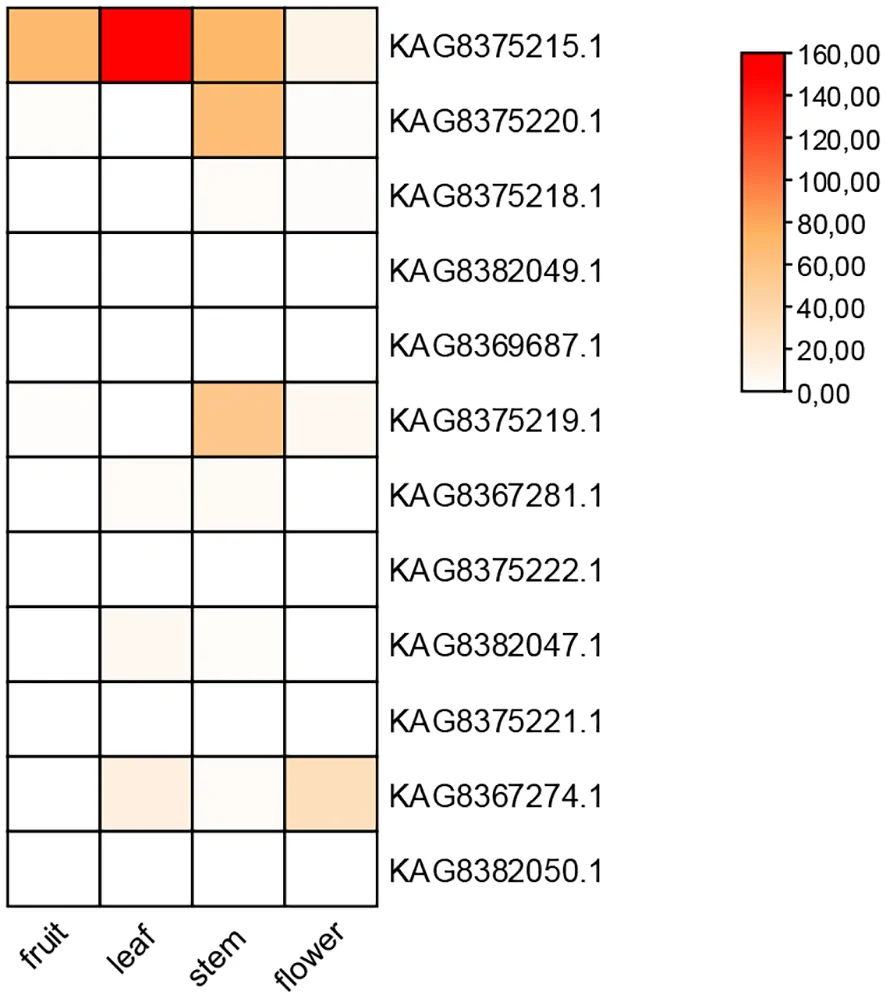
Expression profile of *Buddleja alternifolia CCD4* genes across different tissues. Heatmap showing transcript abundance, expressed as transcripts per million (TPM), for selected *CCD4* genes from *B.alternifolia* in fruit, leaf, stem, and flower tissues. Gene identifiers are shown on the right. Expression values were obtained from RNA-seq datasets available under BioProjectPRJNA577174. The color scale indicates relative expression levels, from low or undetected expression in white to high expression in red.

The homolog of *BdCCD4.2*, encoding KAG8375215, showed the highest expression levels across all organs, particularly in leaves, followed by fruits and stems (**Figure 4**). The genes encoding KAG8375220 and KAG8375219 showed comparatively higher expression in stems, whereas the gene encoding KAG8367274 was most highly expressed in flowers (**Figure 4**). However, because each tissue was represented by a single RNA-seq dataset without biological replication, these patterns should be interpreted as descriptive estimates of relative transcript abundance rather than evidence of statistically supported differential or tissue-specific expression. Consequently, they provide only preliminary indications of possible expression divergence among paralogs and cannot, by themselves, support conclusions regarding subfunctionalization.

### 3.4 Analyses of CCD4 proteins of *B. alternifolia*

All the BaCCD4 proteins were analyzed for the identification of the structural motifs present in the CCD enzymes (**Supplementary Figure S1**). CCDs shares four conserved histidines (His) and three Glu (or less commonly Asp) residues that function cooperatively to coordinate the iron required for activity of these enzymes (Poliakov et al., 2005). The basic CCD fold consists of a 7-bladed β-propeller covered on its top face by a α-dome. The CCD structure possesses fold polarity -a rigid core β-propeller fold juxtaposed with a cluster of non-contiguous α-helical and loop segments of greater conformational flexibility and sequence variability- that is in general useful for the evolution of new activities and substrate specificities (Dellus-Gur et al., 2013). *In silico* analyses were also performed to determine the subcellular location of these proteins (**Table 2**), with 8 proteins predicted to be localized in plastids and 4 in the cytosol, but at low probability.

**Table 2:** Subcellular localization prediction of CCD4 proteins in *B. alternifolia*.

Protein ID	Cytosol	Plastid	Mitochondria	Peroxisome	Soluble	Membrane
KAG8382047.1	0	1	0	0	0.8911	0.1089
KAG8382049.1	0	1	0	0	0.9022	0.0978
KAG8382050.1	0	1	0	0	0.9251	0.0749
KAG8375215.1	0	1	0	0	0.8947	0.1053
KAG8375218.1	0.4290	0.1396	0.1791	0.0979	0.8934	0.1066
KAG8375219.1	0.357	0.1559	0.1691	0.2460	0.8935	0.1065
KAG8375220.1	0.3438	0.1984	0.2136	0.1924	0.8932	0.1068
KAG8375221.1	0.3886	0.3160	0.0424	0.2349	0.8332	0.1668
KAG8375222.1	0	1	0	0	0.8823	0.1177
KAG8369687.1	0	1	0	0	0.9092	0.0908
KAG8367281.1	0	1	0	0	0.8700	0.1300
KAG8367274.1	0	1	0	0	0.8870	0.1130

Subcellular localization was predicted using DeepLoc. The prediction score represents the confidence assigned by the algorithm to the predicted localization, with higher values indicating greater confidence. Scores close to 1.0 indicate strong support for the predicted localization, whereas lower scores suggest greater uncertainty or the possibility of multiple subcellular destinations. Predicted localization provides insights into the potential biological function of CCD4 proteins, as their cellular compartment is closely associated with their role in carotenoid cleavage and apocarotenoid biosynthesis.

### 3.5 Activity assays of selected BaCCD4 enzymes

**Figure 5:**
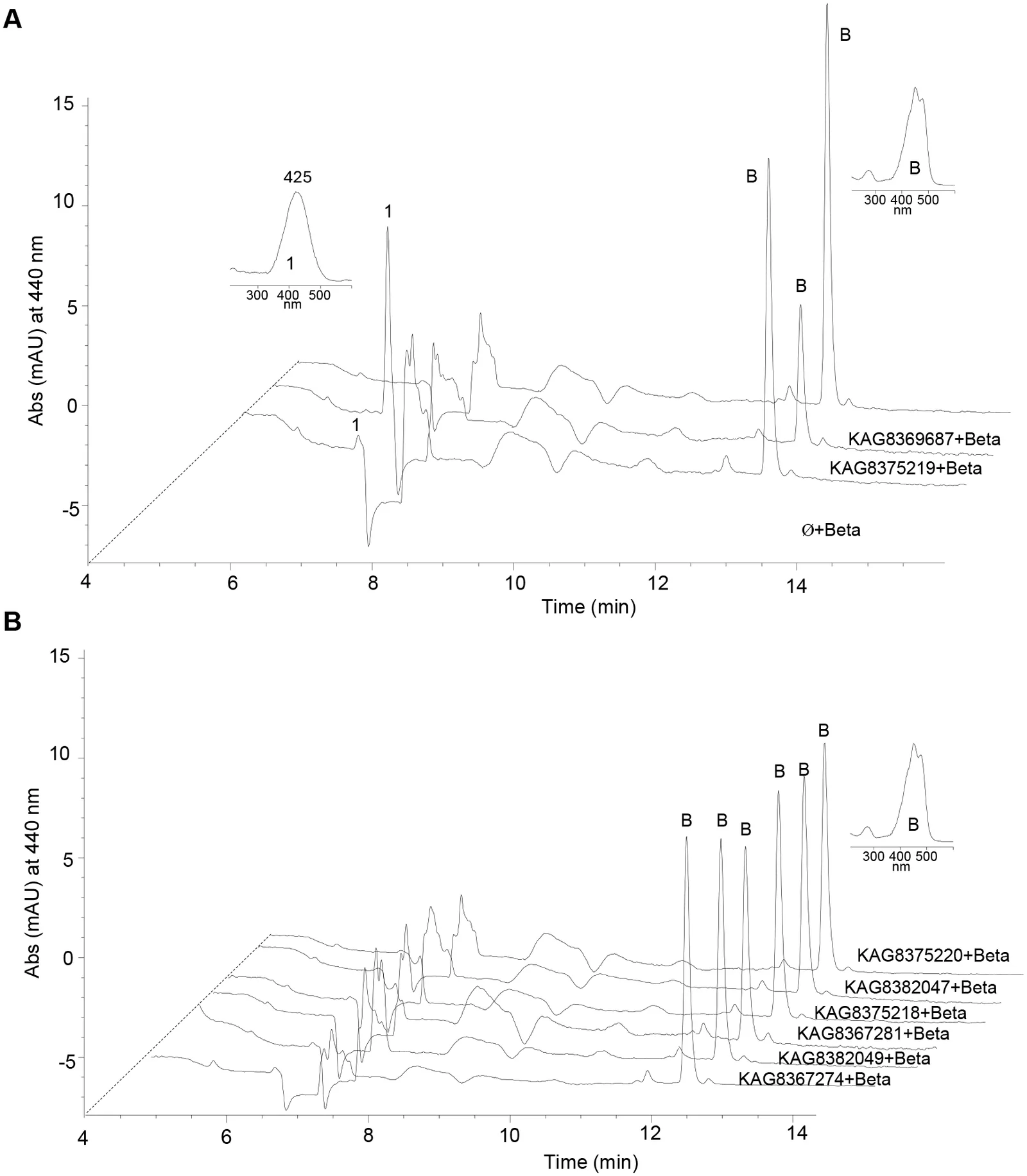
Cleavage activity of *Buddleja alternifolia* CCD4 enzymes toward β-carotene. **(A)** Representative HPLC chromatograms of carotenoid extracts from an *in vivo* assay in which individual CCD4 enzymes were heterologously expressed in β-carotene-producing *Escherichia coli* cells. Expression of KAG8369687 and KAG8375219 CCD4 enzymes resulted in the formation of the apocarotenoid β-apo-12′-carotenal (peak 1). The inset shows the UV-visible absorption spectrum of peak 1, consistent with the spectral characteristics of β-apo-12′-carotenal. **(B)** Representative HPLC chromatograms obtained from β-carotene-producing *E. coli* cells expressing the remaining *B. alternifolia* CCD4 enzymes. No β-carotene-derived apocarotenoid products were detected under the experimental conditions, indicating no detectable cleavage activity toward β-carotene.

**Figure 6:**
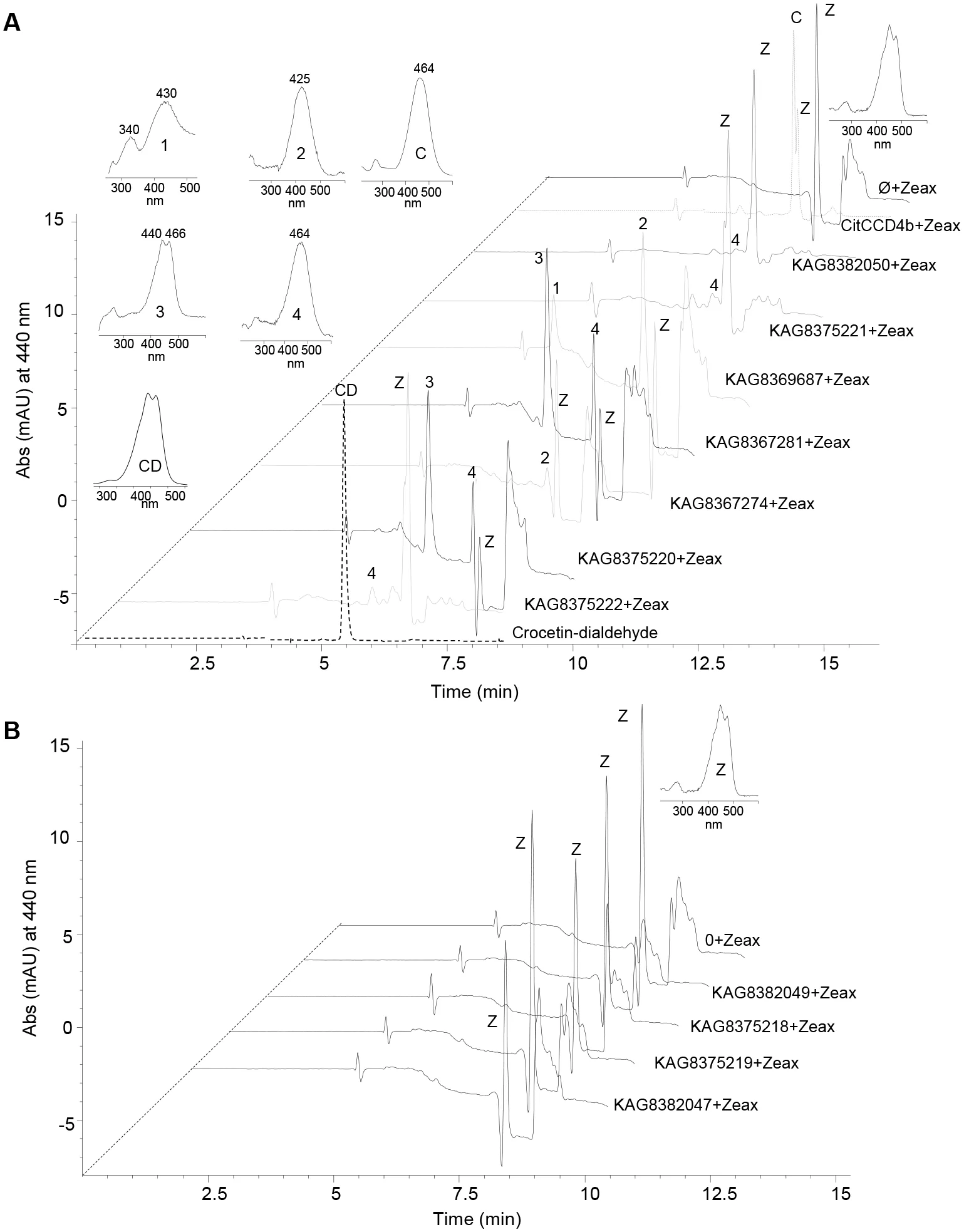
Cleavage activity of *Buddleja alternifolia* CCD4 enzymes toward zeaxanthin. **(A)** Representative HPLC chromatograms of carotenoid extracts from an *in vivo* assay in which individual CCD4 enzymes were heterologously expressed in zeaxanthin-producing *Escherichiacoli* cells. Expression of active enzymes resulted in the formation of several apocarotenoid products: peak1, an unknown apocarotenoid; peak2, 3-hydroxy-β-apo-12′-carotenal; peak3, crocetin dialdehyde; and peak4, 3-hydroxy-β-apo-8′-carotenal. Z denotes the zeaxanthin substrate. HPLC chromatograms of authentic crocetin dialdehyde (CD) and citraurin (C) standards are included. Product identities were assigned by comparison of their retention times and UV-visible absorption spectra with those of the corresponding standards or with previously characterized compounds in the literature. **(B)** Representative HPLC chromatograms of zeaxanthin-producing *E. coli* cells expressing the remaining CCD4 enzymes. No zeaxanthin-derived apocarotenoid products were detected under the experimental conditions, indicating no detectable cleavage activity toward zeaxanthin.

To test whether the BaCCD4 genes encode functional enzymes, we took advantage of a bacteria-based assay (Gomez-Gomez et al., 2020). In these assays, the 11 BaCCD4 enzymes present in the CCD4.1 and in the CCD4.3 cluster were expressed in *E. coli* strains, which contain plasmids to synthesize different carotenoid substrates, including lycopene, β-carotene and zeaxanthin. The accumulation of lycopene confers red color to *E. coli*, while the accumulation of zeaxanthin and β-carotene confer a yellow to orange coloration to the bacteria. Expression of an enzymatically active CCD enzyme will lead to a discoloration of the bacteria because of the cleavage of carotenoid substrates into apocarotenoids. However, determination of the cleavage site will be only determined by the analysis of the resulting products by chromatographic analyses. We employed this assay as outlined under “material and method section”. Bacteria cultures expressing an empty vector were used as a control. Cleavage of the carotenoid lycopene was not observed for any of the BaCCD4 enzymes (**Supplementary Figure S2**). When β-carotene was used as substrate, the extracts from cultures containing KAG8369687and KAG8375219 converted this substrate into product 1 that displayed spectral characteristics of a C12-apocarotenal (λmax 425) (Wild et al., 2006; Sahadevan et al., 2013), suggesting an asymmetric cleavage (**Figure 5A**; **Table 3**), while no cleavage products were identified for the others BaCCD4 enzymes (**Figure 5B**; **Table 3**). The expression of the enzymes in *E. coli* cells expressing zeaxanthin allowed the detection of different products (**Figure 6**; **Table 3**). In the extracts from cells expressing KAG8367281 and KAG8375220 crocetin dialdehyde was detected, peak 3 (λmax 440 and 466 nm) as well as the asymmetric cleavage, peak 4 (λmax 464), identified as β-citraurin (**Figure 6A**; **Table 3**). In the extracts of KAG8369687, when zeaxanthin was tested as substrate, two different products were detected, an unidentified peak 1 (λmax 340 and 430 nm) and peak 2 (λmax 425 nm), which was also detected in the extracts where β-carotene was present, suggesting that most probably this product is the result of the cleavage of β-carotene, identified as β-apo-12′-carotenal (Wild et al., 2006; Sahadevan et al., 2013)(**Figure 5B**; **6A**). However, in the extracts of KAG8367274 the same peak 2 (λmax 425 nm), was detected, but was not observed in the samples of β-carotene. In the extracts of KAG8382050, KAG8375221, and KAG8375222 appeared a unique peak, number 4 (λmax 464), identified as β-citraurin (**Figure 6A**; **Table 3**). The chromatograms obtained for the extracts using the other BaCCD4 enzymes, and where no products were detected using zeaxanthin as substrate are shown in **Figure 6B**; **Table 3**.

**Table 3:** Summary of the substrate specificity and cleavage products of Buddleja alternifolia CCD4 enzymes determined by heterologous expression in carotenoid-producing Escherichia coli.

Enzyme ID	Phylogenetic cluster	Substrate tested	Product detected	Cleavage position	Retention time	λmax (nm)	Identification basis
KAG8367281	CCD4.3	Zeaxanthin	Crocetin dialdehyde Citraurin	7,8/7′,8′ 7,8 or 7′,8′	4.35 5.11	440, 466 464	UV-Vis spectra comparison with standards
KAG8375220	CCD4.3	Zeaxanthin	Crocetin dialdehyde Citraurin	7,8/7′,8′ 7,8 or 7′,8′	4.35 5.11	440, 466 464	UV-Vis spectra comparison with standards
KAG8369687	CCD4.3	β-Carotene	β-Apo-12′-carotenal	12′ or 12	5.03	425	UV-Vis spectra; comparison with reported apocarotenoids
KAG8369687	CCD4.3	Zeaxanthin	Peak 1 β-Apo-12′-carotenal	Asymmetric cleavage	3.54 5.03	340, 430 (Peak 1); 425 (Peak 2)	UV-Vis spectra; comparison with reported apocarotenoids
KAG8367274	CCD4.3	Zeaxanthin	Peak 2	Putative asymmetric cleavage	5.11	425	
KAG8375219	CCD4.3	β-Carotene	C12-apocarotenal	Asymmetric cleavage	5.05	Characteristic of C12-apocarotenal	UV-Vis spectra; comparison with reported apocarotenoids
KAG8375221	CCD4.3	Zeaxanthin	Citraurin	Asymmetric cleavage	5.11	464	UV-Vis spectra comparison with standards
KAG8375222	CCD4.3	Zeaxanthin	Citraurin	Asymmetric cleavage	5.1	464	UV-Vis spectra comparison with standards
KAG8382050	CCD4.1	Zeaxanthin	Citraurin	Asymmetric cleavage	5.11	464	UV-Vis spectra comparison with standards
KAG8382047	CCD4.1	Lycopene, β-carotene, Zeaxanthin	No products detected	No detectable activity	–	–	–
KAG8382049	CCD4.1	Lycopene, β-carotene, Zeaxanthin	No products detected	No detectable activity	–	–	–
KAG8375215	CCD4.1	Lycopene, β-carotene, Zeaxanthin	No products detected	No detectable activity	–	–	–
KAG8375218	CCD4.3	Lycopene, β-carotene, Zeaxanthin	No products detected	No detectable activity	–	–	–

### 3.6 Comparative analysis of crocetin-producing and non-producing highly identity BaCCD4 enzymes

**Figure 7:**
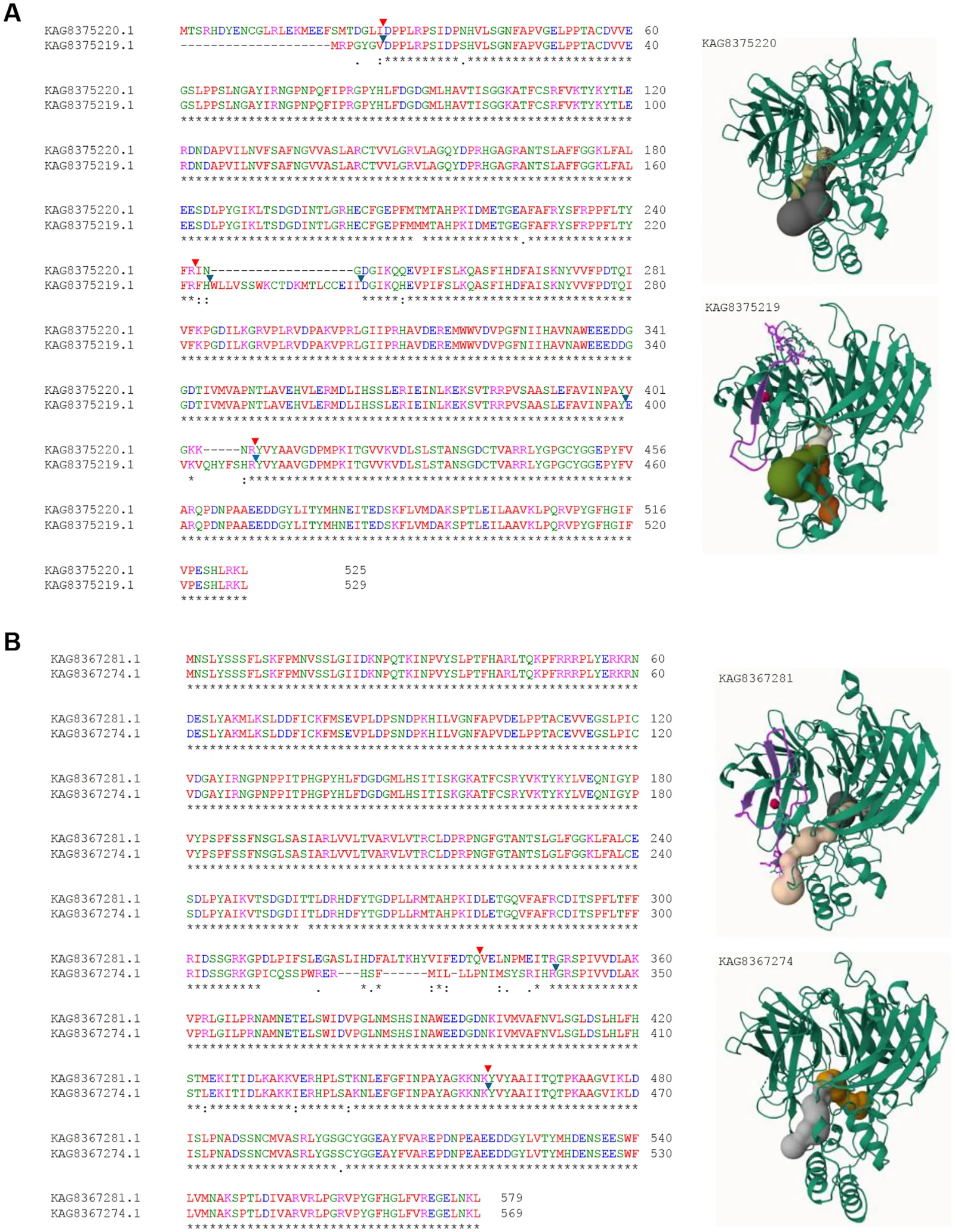
Comparative sequence and structural analysis of *Buddleja alternifolia CCD4* enzymes with high sequence similarity but contrasting enzymatic activities. **(A)** Left, sequence alignment of KAG8375220 and KAG8375219. Arrows indicate intron positions in the corresponding genes (red, KAG8375220; blue, KAG8375219). Amino acid residues that differ between the two proteins are highlighted in pink in the KAG8375219 sequence. Right, predicted three-dimensional structures of KAG8375220 and KAG8375219 showing the putative substrate-access tunnels leading to the catalytic pocket. Residues that differ between the two enzymes are highlighted in pink. **(B)** Left, sequence alignment of KAG8367281 and KAG8367274. Arrows indicate intron positions in the corresponding genes (red, KAG8367281; blue, KAG8367274). Amino acid residues that differ between the two proteins are highlighted in pink in the KAG8367281 sequence. Right, predicted three-dimensional structures of KAG8367281 and KAG8367274, including the putative substrate access tunnels. Differential amino acid residues are highlighted in pink. Sequence alignments were generated using ClustalOmega, and substrate-access tunnels were predicted using MOLE.

To identify the sequence differences between KAG8367274 and KAG8367281, as well as between KAG8375220 and KAG8275219, that may account for the observed differences in enzymatic activity, both amino acid and nucleotide sequences were comparatively analyzed. Enzymes KAG8375220 and KAG8375219 have a 96.44% identity, these sequences showed major differences at amino acid 220 for KAG8375220 and amino acid 242 for KAG8375219 (**Figure 7A**). This difference was associated with the presence of an insertion in KAG8375219, which introduces an additional exon sequence (**Supplementary Figure S3**). Enzymes KAG8367274 and KAG8367281 exhibited 94.9% amino acid sequence identity. The sequence divergence was localized primarily within the region spanning amino acids 310 to 348 (**Figure 7B**). At the nucleotide level, both sequences contained two intronic sequences (**Supplementary Figure S3**). The first nucleotide-level difference affecting the amino acid sequence corresponded to an insertion/deletion (indel) event, which altered the reading frame between the two proteins. This frameshift is responsible for the amino acid changes observed within the 310–348 region and was localized before the first intronic sequence (**Supplementary Figure S3**). The observed changes between KAG8375220 and KAG8375219, and KAG8375220 and KAG8375219 have a clear effect at the level of the tridimensional structure, more specifically modifying the loop close to the tunnel entrance of the substrate (**Figure 7**), which may contribute to the differences observed in activity. Overall changes were also observed at the hydrophobic surfaces and at the internal structure (**Supplementary Figures S4**, **S5**) that may have an impact on the cleavage activities of the enzymes.

## 4 Discussion

Carotenoid cleavage dioxygenase 4 (CCD4) enzymes are central regulators of carotenoid turnover in plants, catalyzing the oxidative formation of apocarotenoids that contribute to pigmentation and specialized metabolism, and display pronounced evolutionary and functional diversification across species (Zheng et al., 2021). The present study provides a comprehensive comparative analysis of the CCD family in *B. alternifolia*, with particular emphasis on the expanded CCD4 subfamily. We simultaneously investigated 11 closely related CCD4 paralogs through integrated genomic, phylogenetic, transcriptional, structural analyses, and functional characterization. This comparative framework allowed us to explore how gene duplication and sequence divergence contributed to the evolution of carotenoid cleavage activities associated with crocin biosynthesis.

### 4.1 Expansion of the *CCD4* family in *B. alternifolia*

The order *Lamiales* comprises 24 families, including *Lamiaceae*, *Gesneriaceae*, *Plantaginaceae*, and *Scrophulariaceae*. The *Scrophulariaceae* is the largest family within the order *Lamiales* (Wang et al., 2024) including species as *Buddleja* and *Verbascum*.

The genome of *B. alternifolia* contains 23 *CCD*-related genes, including representatives of all major *CCD* subfamilies including *CCD1*, *CCD4*, *CCD7*, *CCD8*, *CCD10*, and *NCEDs* (Ahrazem et al., 2016a; Wang et al., 2019; Zhong et al., 2020). Among them, the *CCD4* subfamily underwent an exceptional expansion, with 12 members, which represents the largest *CCD* subgroup identified in this species and in other genus from the order *Lamiales.* Reports in *Paulownia tomentosa* and *Paulownia fortunei* showed 10 and 8 *CCD4* members, respectively (Morote et al., 2023, Morote et al., 2026), 8 *CCD4* genes are also present in the genome of *N. arbor-tristis* (Morote et al., 2025b) and 7 in *B. davidii* (**Figure 3**). Comparative syntenic analyses with *A. trichopoda*, *V. vinifera*, and *B. davidii* suggest that this expansion occurred through lineage-specific duplication events. The chromosomal organization strongly supports recent tandem duplication as a major driver of *CCD4* expansion in *B. alternifolia*. Multiple highly similar genes were clustered on chromosome 10, while another duplicated pair was identified on chromosome 5 and 16. The presence of pseudogenes within the *CCD4*-rich region on chromosome 10 further suggests that this family is undergoing active birth-and-death evolution (Eirín-López et al., 2012). Interestingly, the two pseudogenes were closely related to the gene encoding for KAG8375220, involved in crocetin dialdehyde biosynthesis. Consistent with this evolutionary framework, duplicated *CCD4* genes appear to have undergone functional divergence, as reflected by differences in both transcriptional regulation and cleavage activities.

### 4.2 Evolutionary diversification of crocetin-producing CCD4 enzymes

One of the most significant findings of this study is the functional diversification observed among closely related CCD4 paralogs. The simultaneous biochemical characterization of 11 BaCCD4 enzymes revealed different catalytic behaviors, ranging from proteins without detectable activity under the tested conditions, as the case of KAG8375218, KAG8382047, and KAG8382049, to enzymes capable of producing the asymmetric apocarotenoid cleavage product citraurin, as KAG8382050, KAG8367274, KAG8375221, and KAG8375222. Such activity has been previously reported for a CCD4 enzyme from citrus, responsible for the accumulation of citraurin in the peel of the fruit (Ma et al., 2013; Rodrigo et al., 2013), and more recently in rice, where the CCD4 enzyme regulated mesocotyl elongation and sugar metabolism (Zhang et al., 2026). Particularly noteworthy was the identification of two enzymes, KAG8367281 and KAG8375220, capable of cleaving zeaxanthin to generate crocetin dialdehyde, the immediate precursor of crocetin and crocins. These enzymes belong to two closely related paralogous pairs, KAG8367274/KAG8367281 and KAG8375219/KAG8375220, whose members share more than 94% amino acid identity yet exhibit markedly distinct substrate cleavage profiles. In both pairs, only one paralog retained the capacity to produce crocetin dialdehyde, whereas the corresponding closely related enzyme displayed alternative cleavage activities under the experimental conditions. This pattern strongly suggests that recent duplication events of *BaCCD4* genes were followed by rapid functional divergence, with minimal sequence variation associated with substantial shifts in catalytic specificity. In both enzyme pairs displaying contrasting activities, small sequence alterations, including indels and exon-associated insertions, produced substantial conformational changes in loop regions located near the predicted substrate access channel. Structural modeling suggested that these modifications may alter the architecture surrounding the catalytic cavity, potentially affecting substrate orientation and accessibility. Previous studies have shown that CCD enzymes possess a conserved β-propeller scaffold combined with flexible peripheral regions that contribute to substrate recognition and cleavage specificity (Daruwalla and Kiser, 2020). The occurrence of crocetin-producing activity in two CCD4 paralogs further indicates that the biosynthetic capacity to generate crocin precursors likely emerged through progressive neofunctionalization within the expanded *BaCCD4* family, rather than through a single evolutionary innovation (Copley, 2020). Although the precise evolutionary trajectory cannot be reconstructed from the present data alone, the functional characterization of closely related extant BaCCD4 paralogs differing by only limited sequence changes provides empirical evidence for successive functional divergence following gene duplication. These observations are consistent with, although they do not directly demonstrate, a model of progressive neofunctionalization leading to crocetin-forming activity. Two CCD4 paralogs able to generate crocin are present in *B. davidii* (Ahrazem et al., 2017) and *V. sinuatum* and *V. giganteum* (Morote et al., 2024). Such diversification may reflect selective pressures favoring metabolic plasticity and the spatial or tissue-specific production of crocins.

### 4.3 Tissue-specific expression and metabolic specialization

Expression analyses further supported the functional diversification of the *BaCCD4* family. Distinct paralogs exhibited differentiated expression profiles among organs, suggesting subfunctionalization after duplication. The *BdCCD4.2* homolog KAG8375215 showed strong expression in leaves, indicating a possible role in carotenoid turnover or stress-related apocarotenoid biosynthesis in photosynthetic tissues. The enzymes from other plant species that belong to this cluster showed an asymmetric or symmetric 9,10:9′,10′ cleavage activity (Rubio et al., 2008; Zhang et al., 2026). In contrast, the gene encoding KAG8367274 accumulated preferentially in flowers, where could be involved in aroma production by the generation of 3-OH-β-cyclocitral (Liu et al., 2026). Although KAG8375219 and KAG8375220 showed different cleavage activities, both were predominantly expressed in stems followed by flowers, which presented crocins accumulation in the calix. However, neither crocetin nor crocins have been detected in leaves or stems of Buddleja (Chen et al., 2025), and there are no reports regarding the presence of other apocarotenoid compounds. Nevertheless, we cannot discard the possibility of crocetin being the substrate for KAG8375219 in the plant.

### 4.4 Stepwise evolution of crocetin-producing CCD4 enzymes

The *BaCCD4* family provides an example of functional divergence within a relatively recently expanded, tandemly duplicated *CCD4* cluster, as indicated by the high sequence conservation among paralogs. Our data indicate that *BaCCD4* paralogs encompass a continuum of catalytic behaviors, ranging from inactive or weakly active enzymes to proteins capable of asymmetric zeaxanthin cleavage or partial crocetin-forming activity to fully functional crocetin-producing capacity. This pattern partially resembles the previously characterized *GjCCD4* cluster in *G. jasminoides* (Xu et al., 2020), where four highly similar paralogs displayed markedly different enzymatic properties despite retaining 77-81% amino acid identity. In that system, GjCCD4a was capable of crocin-forming cleavage activity, whereas GjCCD4b and GjCCD4d lacked detectable activity and GjCCD4c showed 9,10 cleave of β-carotene. Together, these observations support the idea that tandemly duplicated *CCD4* genes can undergo substantial biochemical divergence while maintaining high overall sequence conservation. The coexistence of inactive, partially functional, and specialized *BaCCD4* paralogs is consistent with a neofunctionalization model following tandem gene duplication (Birchler and Yang, 2022). Under this framework, duplicated copies may accumulate mutations affecting substrate recognition or cleavage specificity without loss of the ancestral carotenoid cleavage function at the family level. Importantly, the BaCCD4 enzymes do not separate into strictly discrete functional categories. Instead, the presence of enzymes with asymmetric cleavage activity (KAG8367274, KAG8382050, KAG8375221 and KAG8375222) and identities ranging from 51-88% suggest that changes in regioselectivity may occur progressively through several intermediates. In this context, the coexistence within the BaCCD4 family of enzymes producing distinct apocarotenoid profiles may reflect transitional evolutionary intermediates, where asymmetric cleavage activities represent partially optimized catalytic states preceding efficient crocetin production observed for KAG8375220 and KAG8367281.

The repeated recruitment of CCD4 enzymes for crocetin biosynthesis (Ahrazem et al., 2017; Xu et al., 2020; Morote et al., 2024, Morote et al., 2025b) also highlights the broader evolutionary flexibility of carotenoid cleavage dioxygenases in plant specialized metabolism. Because CCD enzymes already act on chemically constrained polyene substrates, relatively small changes affecting substrate orientation or cleavage position may generate distinct apocarotenoid products. In this context, crocetin-forming activity may not require the evolution of an entirely novel catalytic framework, but rather modification of cleavage specificity within an existing enzymatic scaffold. The high sequence conservation observed among BaCCD4 paralogs despite their divergent product profiles is consistent with this interpretation.

## 5 Conclusions

The functional characterization of the BaCCD4 family establishes *B. alternifolia* as a valuable system for investigating the molecular basis of regioselectivity in plant CCD4 enzymes. Future studies integrating mutagenesis, structural modelling, and ancestral sequence reconstruction may help identify the minimal sequence changes required for the transition from canonical carotenoid cleavage to crocetin-forming activity. More broadly, our findings provide evidence that novel specialized metabolic functions can emerge through incremental modification of pre-existing enzymatic activities, establishing B. alternifolia as a promising model for investigating the evolutionary origins of crocetin-producing CCD enzymes.

## Data Availability

The raw data supporting the conclusions of this article will be made available by the authors, without undue reservation.
