# Supplementary material for: Evolutionary adaptation of CCD4 enzymes in Buddleja alternifolia for crocetin biosynthesis
Source: Front Plant Sci. 2026 Aug 20;17:1916011. doi: 10.3389/fpls.2026.1916011 (PMC13538092; doi:10.3389/fpls.2026.1916011)
Supplement: Supplementary file 1 [file DataSheet1.pdf]

Supplementary material

Evolutionary adaptation of CCD4 enzymes in *Buddleja alternifolia* for crocetin biosynthesis

Eduardo Parreño[1,Φ], Lucía Morote[1,2,Φ], Alberto J. López Jiménez[1], Elena Moreno-Giménez[1], Ángela Rubio-Moraga[1], Oussama Ahrazem[1,*], Lourdes Gómez Gómez[1,*]

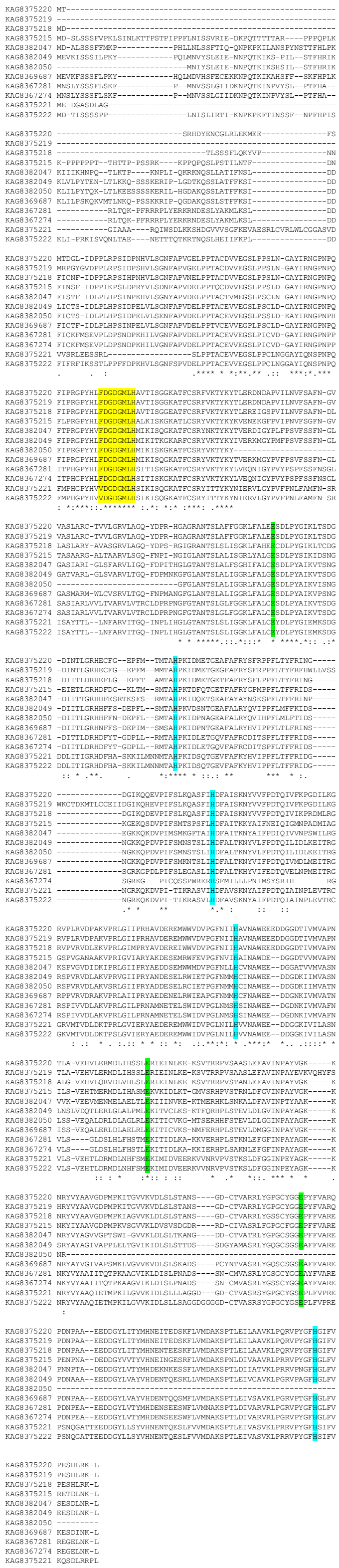

Figure S1. Sequence alignment and topology of CCD sequences from *B. alternifolia*. The conserved iron-binding His and Glu residues are marked in blue and green, respectively. The yellow label indicates residues that appear to be present in active CCDs.

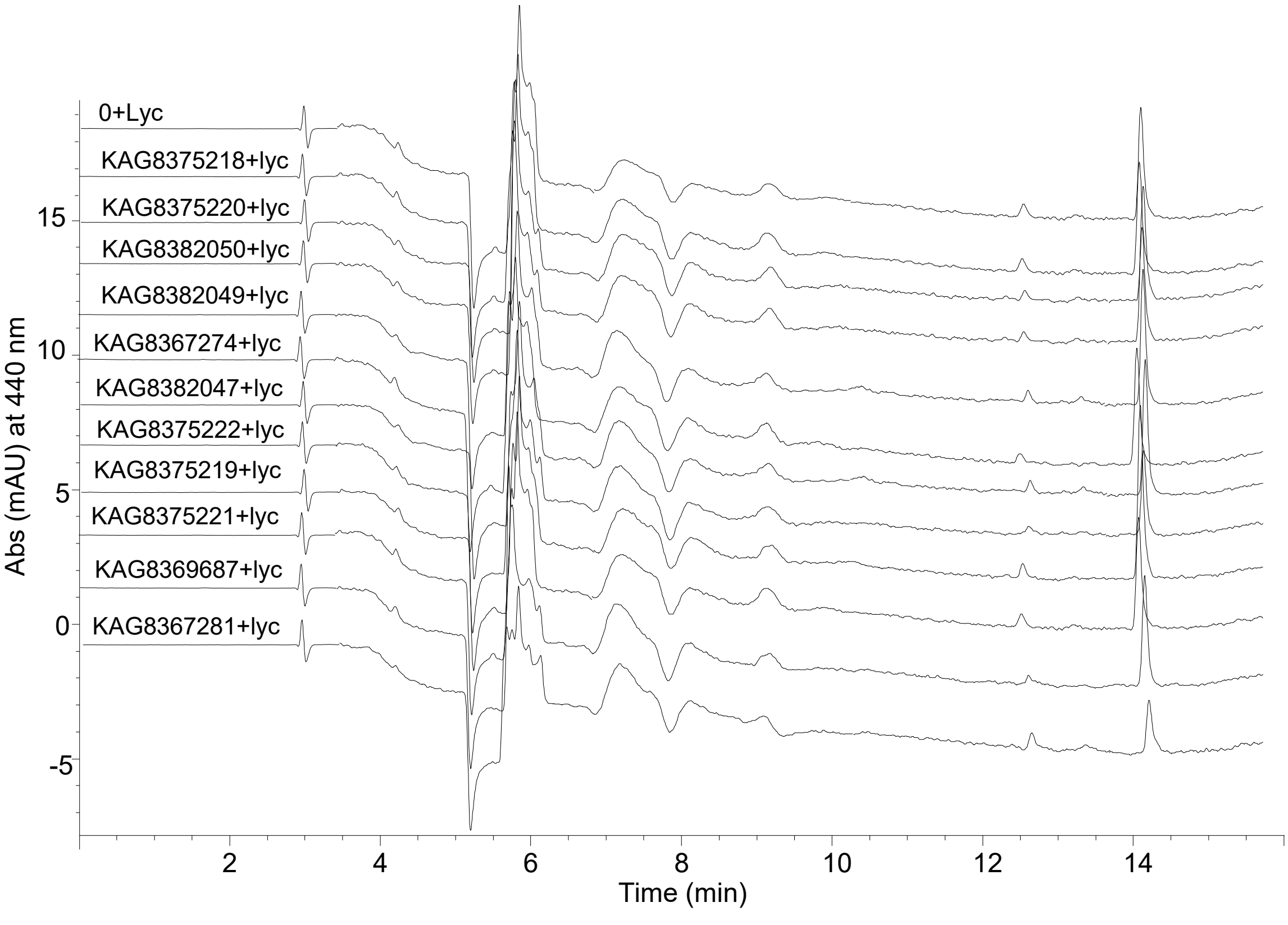

Figure S2. Representative HPLC chromatograms of extracts of reactions of different BaCCD4 enzymes texpressed in E. coli cells that accumulate lycopene.

KAG8275219

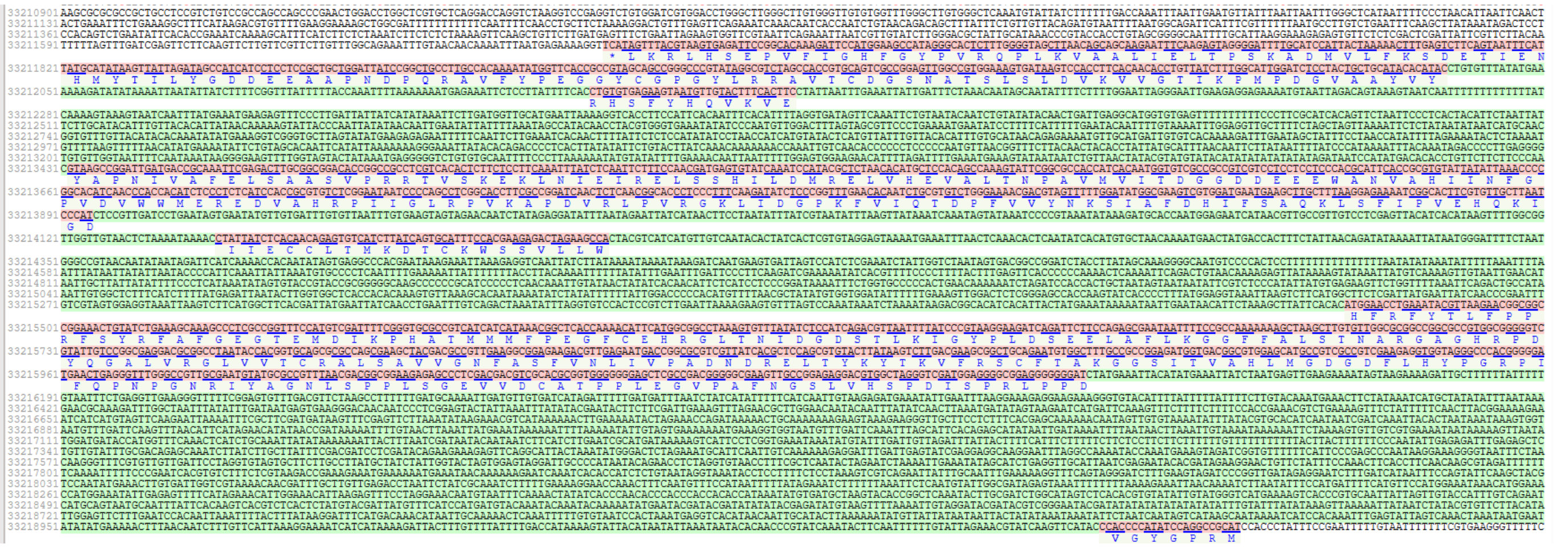

KAG8275220

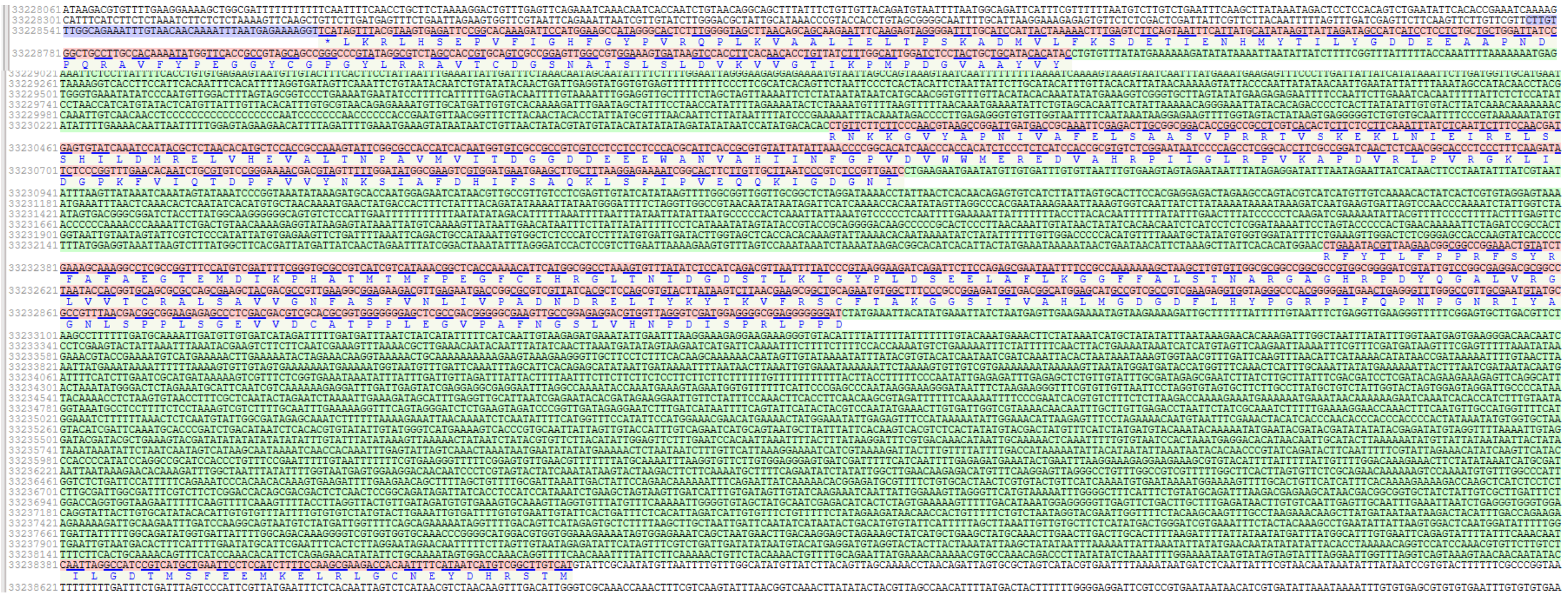

KAG8367274

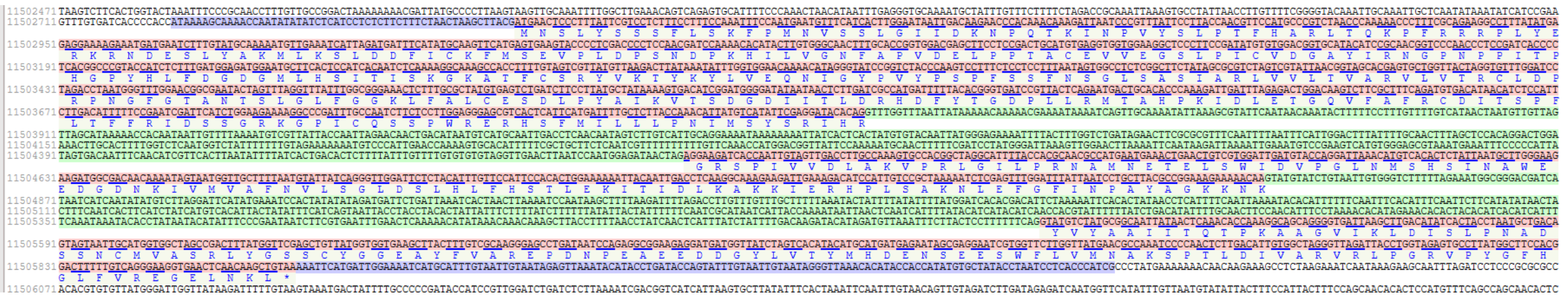

KAG8367281

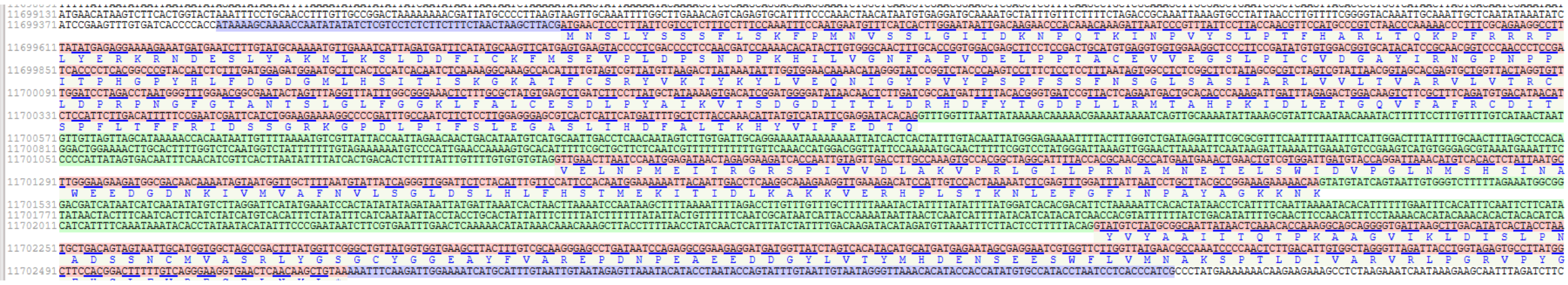

Figure S3. Genomic structure and amino acid sequence of KAG8367274, KAG8367281, KAG8275219 and KAG8275220. In green are highlight the intronic sequences, in pink the exon sequences and in lilac the 5’ and 3’ untranslated sequences.

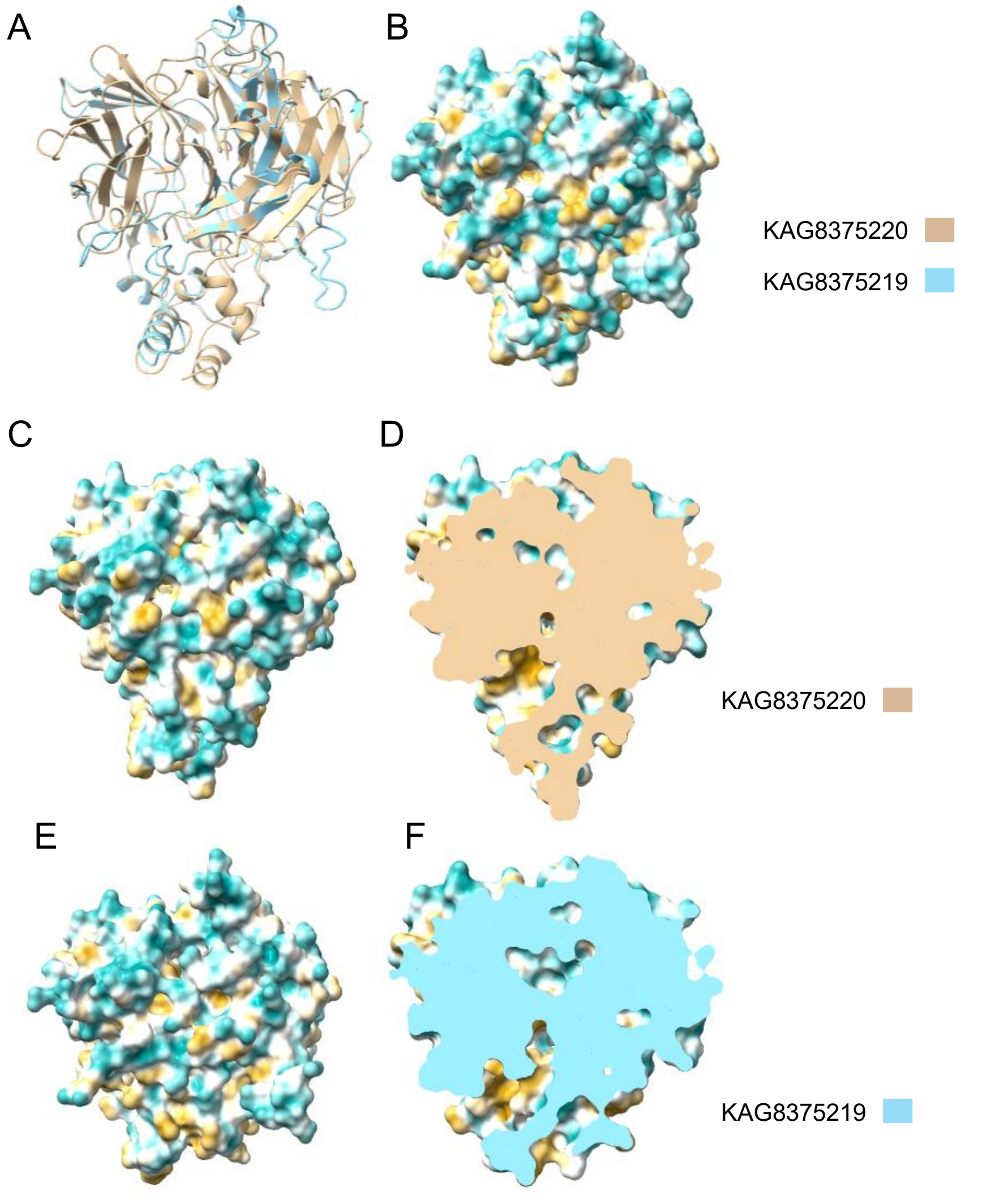

Figure S4. Structural comparisons of KAG8375220 and KAG8375219. A, Global view of the superimposed 3D structures of KAG8375220 and KAG8375219. B, Global view of the superimposed surfaces of KAG8375220 and KAG8375219 colored by the molecular lipophilicity potential, where cyan denotes hydrophilic residues and gold denotes hydrophobic ones. Image C shows KAG8375220 color surface by hydrophobicity and D a close-up sliced view of the tunnel entrance that brings to the active site cavity. Image E shows KAG8375219 color surface by hydrophobicity and F a close-up sliced view of the tunnel entrance that brings to the active site cavity. The UCSF Chimera X program was used for the analyses.

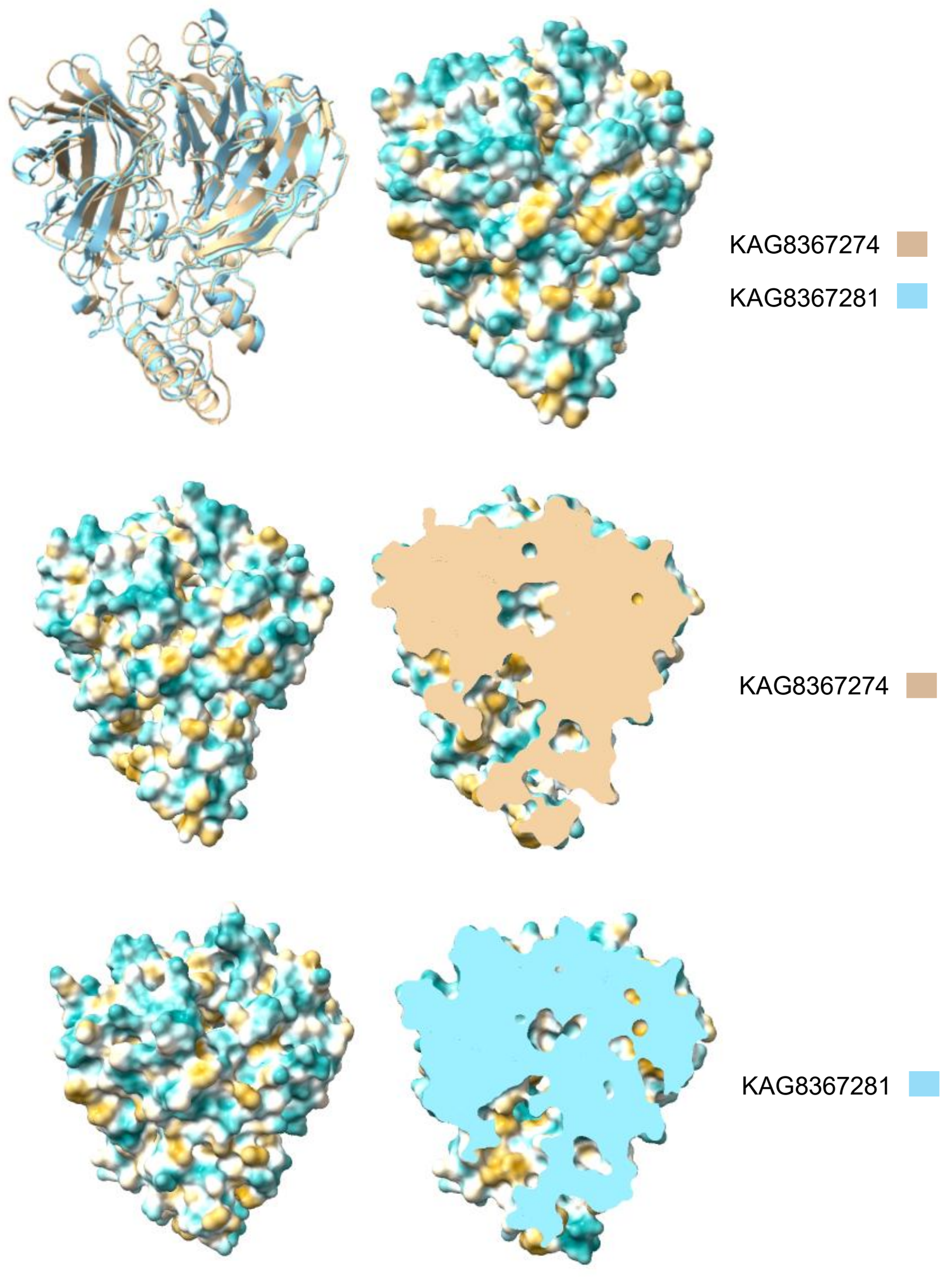

Figure S5. . Structural comparisons of KAG8367274 and KAG8367281. A, Global view of the superimposed 3D structures of KAG8367274 and KAG8367281. B, Global view of the superimposed surfaces of KAG8367274 and KAG8367281 colored by the molecular lipophilicity potential, where cyan denotes hydrophilic residues and gold denotes hydrophobic ones. Image C shows KAG8367274 color surface by hydrophobicity and D a close-up sliced view of the tunnel entrance that brings to the active site cavity. Image E shows KAG8367281 color surface by hydrophobicity and F a close-up sliced view of the tunnel entrance that brings to the active site cavity. The UCSF Chimera X program was used for the analyses.

Table S1. SRA accessions and corresponding BioSamples for RNA-seq datasets from different tissues of Buddleja alternifolia included in BioProject PRJNA577174.

SRA accesion	Biosample	Tissue
SRR10305034	SAMN13022357	Fruit
SRR10305045	SAMN13022356	Leaf
SRR10305056	SAMN13022355	Stem
SRR10305067	SAMN13022354	Flower
